# Process Safety Assessment of the Iron-Catalyzed 1,2-*cis*-Selective Glycal Aminoglycosylation

**DOI:** 10.21203/rs.3.rs-8663578/v1

**Published:** 2026-01-27

**Authors:** Dakang Zhang, Nicola Colombo, Zixiang Jiang, Davide Pirola, Marino Nebuloni, Hao Xu

**Affiliations:** Brandeis University

**Keywords:** Iron, Catalysis, Glycosylation, Process Safety Assessment, 1,2-cis-Aminoglycoside

## Abstract

Complex glycans are directly involved in numerous disease processes and their structural motifs are increasingly incorporated in pharmaceuticals and biological probes. Therefore, the development highly stereoselective and broadly effective glycosylation processes that are safe and readily scalable has been of great value in both academia and industry. We report herein a process safety assessment of an iron-catalyzed, entirely 1,2-*cis*-selective glycosylation process for the synthesis of a broad range of biologically important 1,2-*cis*-aminoglycosides. Differential Scanning Calorimetry analysis of the corresponding catalyst, amination reagents, and an array of representative 1,2-*cis*-aminoglycosides revealed that all of them are thermal stable at temperatures at least 200°C above the glycosylation temperature. Drop Weight Test of the amination reagents suggested that they are impact-stable. Guided by this assessment, we have developed a multigram-scale synthesis of stereochemically pure Tn antigen via the iron-catalyzed 1,2-*cis*-selective glycal aminoglycosylation.

## Introduction

Complex carbohydrates are directly involved in numerous physiological and disease processes, so their structural motifs are increasingly incorporated in pharmaceuticals and biological probes (Figure 1).^1–4^ Over the past century, many powerful chemical glycosylation methods have been invented,^5–9^ but one major hurdle still remains: the stereoselectivity in glycosylation often varies with substrates, so it is difficult to develop a highly stereoselective glycosylation method that is also broadly effective. In particular, there is no general solution for highly 1,2-*cis*-selective glycosylation.^10^

1,2-*cis*-2-Amino glycosidic linkages are present in many pharmaceuticals and biologically important complex glycans, including anticoagulant heparan sulfate^11–12^ and Fondaparinux^1^, aminoglycoside antibiotics, and *O*-linked glycopeptides. Unlike 1,2-*trans*-2-amino glycosides which can be prepared by established methods,^13–20^ it is still challenging to reliably form 1,2-*cis*-2-amino glycosidic linkages in high stereoselectivity. The existing *cis*-selective methods leverage non-neighboring-participating groups, particularly the azido group, to enhance the 1,2-*cis*-selectivity (Figure 2A);^21–31^ however, these methods are most effective for certain types of substrates with specific substituents or conformations. Structural variations in either glycosyl donors or glycosyl acceptors often compromise the high 1,2-*cis*-selectivity. In addition, organic azides may present potential safety concerns for their handling, which adds to the difficulty for process development of large-scale 1,2-*cis*-selective aminoglycosylation.

Inspired by our research program of the iron-catalyzed selective nitrogen atom transfer for olefin functoinalization,^32–34^ we envisioned a unique approach that would lead to exclusively 1,2-*cis*-selective aminoglycosylation (Figure 2B). We hypothesized that an iron catalyst could activate a glycosyl acceptor and an amination reagent, and subsequently transfer both moieties to a glycal that is otherwise unreactive in the absence of the catalyst. As both the nitrogen atom transfer step and the glycosyl-acceptor transfer step would be directed by the iron catalyst, we predict that the amido group and the catalyst-activated glycosyl acceptor could be cooperatively delivered to a glycal in an exclusively *cis*-selective manner. Distinct from existing glycosylation methods, the unactivated glycosyl acceptor is unlikely to compete for the same reactive intermediate, which should minimize or even eliminate stereochemical erosion from the non-stereoselective pathways.

Directly building upon a pair of iron-catalyzed olefin aminohydroxylation reactions discovered in our lab in 2013^32^ and 2014^33^ (Figure 3A), we have recently developed an iron-catalyzed 1,2-*cis*-selective glycal aminoglycosylation method (Figure B).^35^ A readily available iron catalyst–Fe(**L1**)(BF_4_)_2_(MeCN)(H_2_O)_2_ **1**^36^, is uniquely effective in promoting the exclusively 1,2-*cis*-selective aminoglycosylation between a bench-stable glycal, a glycosyl acceptor, and an amination reagent (Figure 3B). Catalyst **1** facilitates facile cleavage of the N-O bond of acyloxy carbamate **2** and transfers both the carbamate fragment of **2** and the glycosyl acceptor to the glycal in an exclusively *cis*-selective manner from the a-face, affording the desired aminoglycoside (*dr* >20:1). This 1,2-*cis*-selective method is effective for a broad range of glycosyl donors and acceptors (Figure 4). Additionally, it plays a pivotal role in highly stereoselective synthesis of biologically important Tn antigens^37–39^ and heparan sulfate oligosaccharides^40^ (Figure 3B).

The iron catalyst **1** capitalizes on the high energy N–O bond in acyloxy carbamate **2** to drive the glycosylation under cryogenic conditions (−40 °C); however, it is known that thermal hazards could be present in molecules with weak N–O bonds,^41^ which may present potential safety concerns for their handling, with regard to their thermo- and mechanical impact stabilities. In order to explore the feasibility of this iron-catalyzed glycosylation on an industrial scale, it is imperative to carry out chemical hazard assessment of this reaction. A reactive chemical hazard assessment refers the identification and possibly quantification of dangerous energy release scenarios for a chemical process of interest. Herein, we describe our chemical safety studies and potential hazard assessment of the iron-catalyzed 1,2-*cis*-selective glycosylation, using Differential Scanning Calorimetry (DSC) and Drop Weight Test (DWT) analysis.

## Results and Discussion

We selected the iron-catalyzed glycosylation of primary and secondary acceptors (**4** and **6**) with glycal **3** and amination reagents **2a/2b** as the model reactions for safety analysis. Kinetic studies of the glycosylation of primary acceptor **4** with electron-rich glycal **3** and amination reagent **2a** (Eq. 1 in Scheme 1) suggested that its initial rate has a *first-order* dependence on the iron catalyst, an *inverse first-order* dependence on acceptor **4**, and a *zero-order* dependence on both amination reagent **2a** and glycal **3**.^35^ Kinetic studies further revealed that the initial rate for glycosylation of secondary acceptor **6** with amination reagent **2b** and glycal **3** (Eq. 2 in Scheme 1) has a *first-order* dependence on the iron catalyst but a *zero-order* dependence on each of acceptor **6**, glycal **3**, and amination reagent **2b**.^35^

As our first step to explore the feasibility of applying this reaction on an industrial scale, we conducted a series of thermal stability tests using Differential Scanning Calorimetry (DSC) (Fig. 5, Fig. 6, Fig. 7, and Figure S13).

We first evaluated the thermal stability of amination reagents **2a** and **2b** as they contain high energy N–O bonds and are used in super-stoichiometric amounts in the glycosylation. The DSC experiment concluded that amination reagent **2a** melts at 77°C and starts to decompose at 122°C (T_onset_: 154°C, ΔH: −637 J/g) (Fig. 5). A second exothermic event was observed starting around 188°C (T_onset_: 190°C, ΔH: −354 J/g), which is likely due to decomposition of the 2-bromoisobutyric acid moiety in **2a**. Amination reagent **2b**, derived from 2-acetoxyisobutyric acid, melts at 62°C and shows only one exothermic event (T_onset_: 172°C, ΔH: −610 J/g) in the thermogram (Fig. 5). Notably, the decomposition onset temperatures of both **2a** and **2b** are significantly higher than the glycosylation temperature (− 40°C), providing a safety operating temperature margin (> 200°C) for large-scale operation.

The *N*-Boc group is known to undergo spontaneous decarboxylation at elevated temperatures;^42^ therefore, we next evaluated the thermal stability of the corresponding *N*-Troc–protected amination reagents **2d** and **2e** (Fig. 5). Notably, **2d** and **2e** are much less-efficient for the glycal 1,2-*cis*-aminoglycosylation which provides low conversions and low yields.^35^ Surprisingly, both **2d** and **2e** exhibited markedly broader exothermic events in their DSC thermograms, spanning more than 130°C, which were accompanied by substantially larger enthalpies of decomposition (ΔH: −1232 and − 1782 J/g, respectively). These distinct thermal profiles suggested that the *N*-Boc-protected acyloxy carbamates (**2a** and **2b**) are thermally more stable than their *N*-Troc-protected analogs and that the presence of multiple halogen atoms may contribute to their decreased thermal stability.

Our kinetic studies of this glycosylation suggested that its initial rate has a *first-order* dependence on iron catalyst **1**, so we further evaluated the catalyst’s thermal stability. We observed that iron catalyst **1** is thermally stable and there is no exothermic event up to 143°C in an isochoric setting (T_onset_: 153°C, ΔH: −293 J/g, Fig. 6). Interestingly, two endothermic events were observed in an isobaric setting (T_onset_: 137°C and ΔH: 196 J/g, T_onset_: 179°C and ΔH: 128 J/g), presumably due to the decomposition of **1** with the development of gaseous products. Once again, the decomposition onset temperature of **1** is much higher than the catalytic glycosylation temperature, which offers a comfortable temperature margin for safe large-scale operation.

Based upon these DSC results, we subsequently evaluated the mechanic impact sensitivities of iron catalyst **1** and amination reagents (**2a** and **2b**) by the Fall Hammer Test (Drop Weight Test). Gratifyingly, all of them uniformly demonstrate negative results in the DWT. These data confirm higher stability of the product under any mechanical impact during the process product manipulation.

To assure that the 1,2-*cis*-aminoglycosylation products are amenable to large-scale production, we also carried out the DSC analysis of two 1,2-*cis*-aminoglycosides (**5** and **7**) that were used in kinetic studies (Fig. 7). Aminoglycoside **5** melts at 156°C, yet its DSC thermogram displays a couple of mild endothermic and exothermic events up to 198°C (ΔH: up to − 24 J/g), presumably due to solid-solid transition. We observed a significant thermal decomposition starting at 215°C (T_onset_: 252°C, ΔH: −110 J/g). Interestingly, aminoglycoside **7** displays only an exothermic event at a higher temperature in the DSC analysis (T_onset_: 240°C, ΔH: −145 J/g). These results suggested that the 1,2-*cis*-aminoglycosylation products are thermally stable for handling at room temperature. Furthermore, we carried out the DSC experiments for several other synthetically valuable aminoglycosides with distinct steric and electronic properties, including **8, 9**, and **10** and concluded that they are all thermal stable (Fig. 7).

We further explored potential runaway scenarios in which one or more components were inadvertently omitted from the iron-catalyzed glycosylation (Scheme 2). We observed that omission of a glycosyl acceptor resulted in formation of a 1,2-*cis*-glycal aminoacyloxylation product **11**. In DSC analysis of **11**, only a single exothermic event was observed (T_onset_ = 190°C, ΔH: −256 J/g, Fig. 7). In the absence of a glycal donor, regardless the presence of a glycosyl acceptor **4**, amination reagent **2a** was partially decomposed to BocNH_2_ (**12**) and 2-bromoisobutyric acid (**13**) with low conversion (< 20% conversion). DSC analysis of **12** revealed two endothermic events (T_onset_ = 88°C, ΔH = 22 J/g; T_onset_ = 228°C, ΔH = 230 J/g), in addition to its melting at 108°C (Figure S13). Collectively, these results suggested that the iron-catalyzed glycal 1,2-*cis*-aminoglycosylation does not exhibit hazardous exothermic behavior under plausible reagent-omission scenarios and that this glycosylation is amenable to large-scale operation.

The observed thermal stability of 1,2-*cis*-amido galactosyl serine benzyl ester **10** (Tn antigen) is particularly encouraging, as it is a key building block for *O*-linked glycopeptide synthesis. The synthesis of stereochemically pure Tn antigens is critically important for the access to homogeneous *O*-linked glycopeptides. However, this has been challenging because the embedded 1,2-*cis*-amidoglycosidic linkage is difficult to form reliably in high stereoselectivity. The vast majority of existing syntheses of Tn antigens leverage non-neighboring participating groups, most notably the azido group, to enhance the *cis*-selectivity.^10, 21–25, 30, 43–54^ However, structural variations either in galactosyl donors or amino acid acceptors often compromise the high 1,2-*cis*-selectivity, affording diastereomeric mixtures.^21, 29, 51–64^ Based upon this process safety assessment, we developed a 10 mmol scale, iron-catalyzed 1,2-*cis*-aminoglycosylation procedure for the synthesis of fully protected Tn antigen (**10**). The commercially available and bench-stable tri-*O*-acetyl-D-galactal (**14**) and *N*-Cbz serine benzyl ester (**15**) are readily glycosylated by iron catalyst **1** and amination reagent **2a**, affording protected Tn antigen **10** in high yield (Scheme 3, 6.09 g, 85% yield). Rapid and scalable postglycosylation deprotection procedures minimize aqueous workups and readily afford single diastereomeric Tn antigen (**16**).^37^

## Conclusions

In conclusion, we have carried out process safety assessment of the iron-catalyzed glycal 1,2-*cis*-aminoglycosylation process through Differential Scanning Calorimetry and Drop Weight Test of the corresponding iron catalyst, reagents, and representative glycosylation products. These experiments suggest that all of them are thermal stable at temperatures at least 200°C above the glycosylation temperature and that the amination reagents are impact-stable. Guided by these process safety studies, we have developed a multigram-scale, iron-catalyzed glycosylation process that readily affords stereochemically pure Tn antigen.

## Experimental Section

### Differential Scanning Calorimetry (DSC).

The DSC measurements were performed in a Mettler Toledo DSC1 using 40 μL aluminum punctured crucibles under nitrogen atmosphere or 75 μL medium pressure Perkin Elmer stinless steel crucibles under air atmosphere. All measurements were carried out at a heating rate of 5 K/min.

### Mechanical Sensitivity Test.

The Fall Hammer Test (Drop Weight Test) was designed to determine the sensitivity of potentially high explosive compounds and it was carried out by dropping a 7 Kg-weight from the height of 0.5 m in accordance to the UN Recommendation on the Transport of Dangerous Goods, Manual of Tests and Criteria–Test 3 (a) (ii) as well as EN 13631–4.

### A Procedure for the Large-Scale Iron-Catalyzed Glycal 1,2-*cis*-Aminoglycosylation of 15 with 14 and 2a.

To a flame-dried 100 mL round bottom flask (flask **A**) equipped with a stir bar were added tri-*O*-acetyl-D-galactal (**14**) (2.72 g, 10 mmol, 1.0 equiv), *N*-Cbz serine benzyl ester (**15**) (4.61 g, 14 mmol, 1.4 equiv), iron catalyst Fe(**L1**)(BF_4_)_2_(MeCN)(H_2_O)_2_ (**1**) (580 mg, 1 mmol, 10 mol %), and freshly activated 5 Å powdered molecular sieves (*ca.* 4 g). After the flask was evacuated and backfilled with N_2_ twice, anhydrous CH_2_Cl_2_ (8 mL) and freshly opened 1,4-dioxane (2 mL) were added and the flask was cooled to − 78°C. To a flame-dried 25 mL round bottom flask (flask **B**) was added acyloxyl carbamate **2a** (4.23 g, 15 mmol, 1.5 equiv). Flask **B** was evacuated and backfilled with N_2_ twice and anhydrous CH_2_Cl_2_ (10 mL) was added. Then the solution in flask **B** was transferred to flask **A** via a syringe in 10 min. The reaction was kept at − 78°C for an additional 3 min before switched to − 40°C. The reaction was kept at − 40°C for 4 h and quenched by precipitating the iron catalyst with Et_2_O (40 mL) at the same temperature. The mixture was stirred for two minutes and subsequently warmed up to room temperature. The solution was then filtered through a short pad of Celite^®^ and washed with saturated aq. NaHCO_3_ solution (15 mL). The organic phase was separated from the aqueous one, which was further extracted with CH_2_Cl_2_ (15 mL × 3). The combined organic phase was dried over anhydrous Na_2_SO_4_ and concentrated *in vacuo*. The residue was purified through a silica gel flash column (hexanes/acetone: from 20:1 to 5:1) to afford the desired product 10 as white foam (6.09 g, 85% yield).

## Supplementary Material

Experimental procedure, characterization data for all new compounds and selected NMR spectra. The Supporting Information is available free of charge on the ACS Publications website.

Schemes 1 to 3 are available in the Supplementary Files section.

## Data Availability

All data generated or analyzed during this study are included in this published article and its Supporting Information file.
